# A Case of a Central Embolic Shower in a Patient With Likely Pancreatic Malignancy

**DOI:** 10.7759/cureus.105590

**Published:** 2026-03-21

**Authors:** Lauren Wilson, Raza Moosvi

**Affiliations:** 1 Hospital Medicine, Maidstone and Tunbridge Wells NHS Trust, Kent, GBR; 2 General Surgery, Maidstone and Tunbridge Wells NHS Trust, Kent, GBR

**Keywords:** angiogram, arterial thromboembolism, case report, embolic shower, malignancy, non-bacterial thrombotic endocarditis, pancreatic adenocarcinoma (pdac), pancreatic cancer, thromboembolism, trousseau’s syndrome

## Abstract

Systemic malignancy is a well-established driver of hypercoagulability, often manifesting as localised venous or arterial thromboembolism. While pulmonary embolism and stroke are commonly seen, a central embolic shower is an infrequent and often catastrophic presentation. This case highlights a massive embolic event in the presence of suspected pancreatic malignancy.

A 75-year-old female presented with acute-onset lower limb ischaemia in the background of suspected pancreatic malignancy. A CT angiogram revealed not only peripheral arterial occlusion but also concomitant renal and splenic infarcts. Despite management with anticoagulation, the patient's condition deteriorated rapidly, leading to the patient's death.

While central embolic showers are classically pathognomonic for cardiac myxomas, this case demonstrates their association with solid organ malignancies. Clinicians should maintain a high index of suspicion for malignancy in patients presenting with multiorgan infarcts, and this may serve as a marker of advanced, aggressive disease.

## Introduction

The association between malignancy and a hypercoagulable state is well-documented, with thromboembolism serving as a frequent and often debilitating complication [1]. While venous thromboembolism is common, arterial thromboembolism represents a distinct clinical entity. The incidence of arterial thromboembolism is doubled in patients with solid organ malignancies compared to the general population [2]. Furthermore, the occurrence of arterial thromboembolism in patients with cancer is a significant predictor of mortality, associated with poor prognosis and a three-fold increased risk of death [3].

Embolic showers are a recognised but rare phenomenon most often associated with atrial myxomas and other malignancies (e.g., lung sarcoma), where the tumour itself may invade by atrial extension [4].

This report presents a case of an embolic shower in the context of likely pancreatic malignancy, notably occurring in the absence of direct cardiac invasion or primary cardiac pathology. The objective of this case is to highlight that solid organ tumours can trigger multi-site arterial infarctions through systemic coagulopathy alone. This highlights the importance for the clinician to consider occult malignancy in the differential diagnosis of systemic embolic events.

## Case presentation

A 75-year-old female presented to the emergency department with acute, severe epigastric pain. This presentation occurred against a three-month clinical history of progressive right-sided abdominal pain, nausea, and chronic fatigue. She had no previous clinical investigation or examination during this three-month period. Significant constitutional symptoms were noted, including anorexia and unintentional weight loss exceeding 6 kg.

On physical examination, the patient appeared cachectic, pale, and diaphoretic. While cardiovascular and respiratory examinations were unremarkable, abdominal assessment was significant for board-like rigidity and signs of localised peritonitis. Vital signs demonstrated tachycardia but were otherwise unremarkable (Table 1). The patient had no significant past medical history, took no regular medications, and had no significant family history.

**Table 1:** Observations on admission.

Vital sign	Value
Respiration rate	19
Oxygen saturation (%)	96
Heart rate (beats/min)	114
Blood pressure (mmHg)	114/78
Temperature (°C)	36.6

Initial laboratory investigations were significant for a marked inflammatory response, characterised by a leukocytosis of 24.96 x 10^9^/L and C-reactive protein (CRP) of 37 mg/L. Liver function tests (LFTs) were mildly deranged with a bilirubin of 22 µmol/Land alkaline phosphatase of 590 IU/L, while serum amylase remained within normal limits (Table 2).

**Table 2:** Laboratory test results on admission.

Test	Result	Reference value
C-reactive protein (CRP)	37 mg/L	<5 mg/L
Serum amylase	30 U/L	30-118 U/L
Total bilirubin	22 µmol/L	0-21 µmol/L
Alkaline phosphatase (ALP)	590 IU/L	30-130 IU/L
Alanine transaminase (ALT)	58 IU/L	0-40 IU/L
Sodium	139 mmol/L	133-146 mmol/L
Potassium	3.5 mmol/L	3.5-5.3 mmol/L
Creatinine	101 µmol/L	45-84 µmol/L
Haemoglobin	153 g/L	115-165 g/L
White blood cells	24.96 x 10*⁹/L	4.0-10.0 x 10*⁹/L
Platelet count	80 x 10*⁹/L	150-410 x 10*⁹/L
Neutrophils	23.59 x 10*⁹/L	2.0-7.0 x 10*⁹/L
Lymphocytes	0.56 x 10*⁹/L	1.0-3.0 x 10*⁹/L
Monocytes	0.73 x 10*⁹/L	0.2-1.0 x 10*⁹/L
Eosinophils	0.01 x 10*⁹/L	0.0-0.5 x 10*⁹/L
Basophils	0.07 x 10*⁹/L	0.0-0.1 x 10*⁹/L

**Figure 1:**
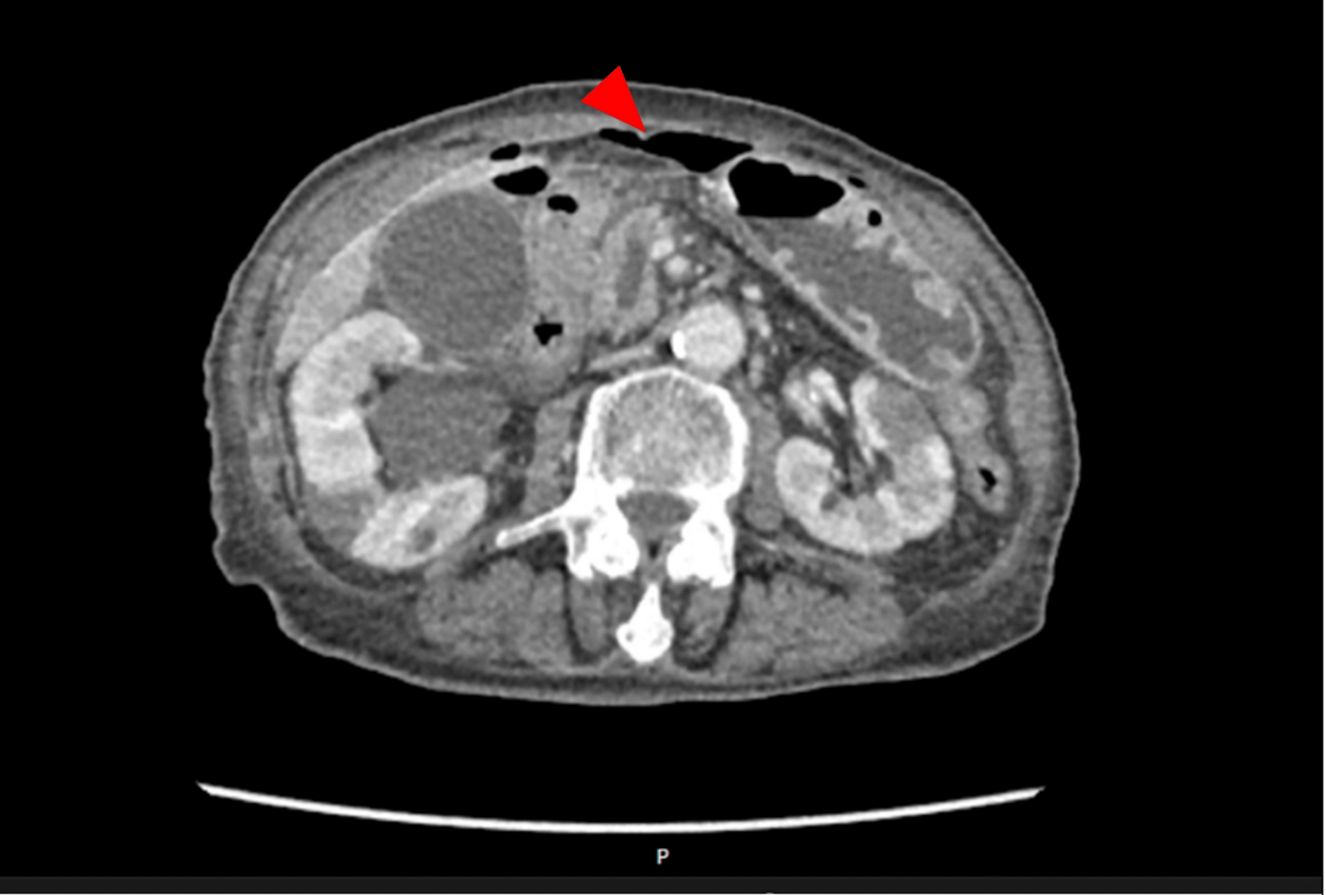
CT of the abdomen and pelvis showing free subdiaphragmatic gas (red arrow).

Urgent computed tomography (CT) of the abdomen and pelvis demonstrated extraluminal gas, highly suggestive of gastrointestinal perforation, likely localised to the duodenum (Figure 1). Notably, the imaging also revealed an ill-defined pancreatic head with associated pancreatic duct dilatation. These findings, in the context of the patient's constitutional symptoms, were highly suspicious for an underlying pancreatic malignancy and warranted further diagnostic correlation.

Following discussion with the patient and her family, she underwent an emergency laparotomy. Intraoperative findings confirmed four-quadrant peritonitis secondary to the suspected perforation and revealed a pancreatic mass. It was unclear if the perforation was related to the pancreatic mass or a coincidental finding. Biopsies were not taken at the time due to extensive faecal contamination within the abdomen. An omental patch repair of the duodenal defect was performed. Postoperative serum tumour markers were significantly elevated with cancer antigen 19-9 (CA 19-9) at >100,000 kU/L, carcinoembryonic antigen (CEA) at 642 ​​​​ug/L*,* and cancer antigen 125 (CA 125) at 1827 U/mL, strongly supporting a diagnosis of pancreatic malignancy.

The patient's postoperative recovery was protracted, marked by significant cachexia, fatigue, and poorly controlled pain. She remained an inpatient with ongoing clinical care and physiotherapy to manage these symptoms. During this time, she remained on prophylactic low-molecular-weight heparin (LMWH), which was initiated on day two postoperatively. Her renal function improved with a return to a baseline creatinine of 47 µmol/L*. *The patient began to improve clinically, and discharge planning was initiated. She was referred to the upper gastrointestinal multidisciplinary team for further investigation of the pancreatic mass.

On postoperative day 18, she reported an acute onset of bilateral lower limb pain, which was more pronounced on the right. Physical examination revealed that the right foot was cool to the touch, with visible cyanosis and digital ischaemia affecting the distal phalanx. Pedal pulses were absent. Simultaneously, laboratory investigations demonstrated a sharp decline in renal function, triggering an acute kidney injury (AKI) stage 2 and worsening LFTs (Table 3).

**Table 3:** Laboratory test results at the time of the embolic event.

Test	Result	Reference value
C-reactive protein (CRP)	296 mg/L	<5 mg/L
Total bilirubin	51 µmol/L	0-21 µmol/L
Alkaline phosphatase (ALP)	1132 IU/L	30-130 IU/L
Alanine transaminase (ALT)	107 IU/L	0-40 IU/L
Sodium	133 mmol/L	133-146 mmol/L
Potassium	4.4 mmol/L	3.5-5.3 mmol/L
Creatinine	100 µmol/L	45-84 µmol/L
Haemoglobin	83 g/L	115-165 g/L
White blood cells	24.74 x 10*⁹/L	4.0-10.0 x 10*⁹/L
Platelet count	152 x 10*⁹/L	150-410 x 10*⁹/L
Neutrophils	22.49 x 10*⁹/L	2.0-7.0 x 10*⁹/L
Lymphocytes	0.90 x 10*⁹/L	1.0-3.0 x 10*⁹/L
Monocytes	1.24 x 10*⁹/L	0.2-1.0 x 10*⁹/L
Eosinophils	0.01 x 10*⁹/L	0.0-0.5 x 10*⁹/L
Basophils	0.10 x 10*⁹/L	0.0-0.1 x 10*⁹/L

**Figure 2:**
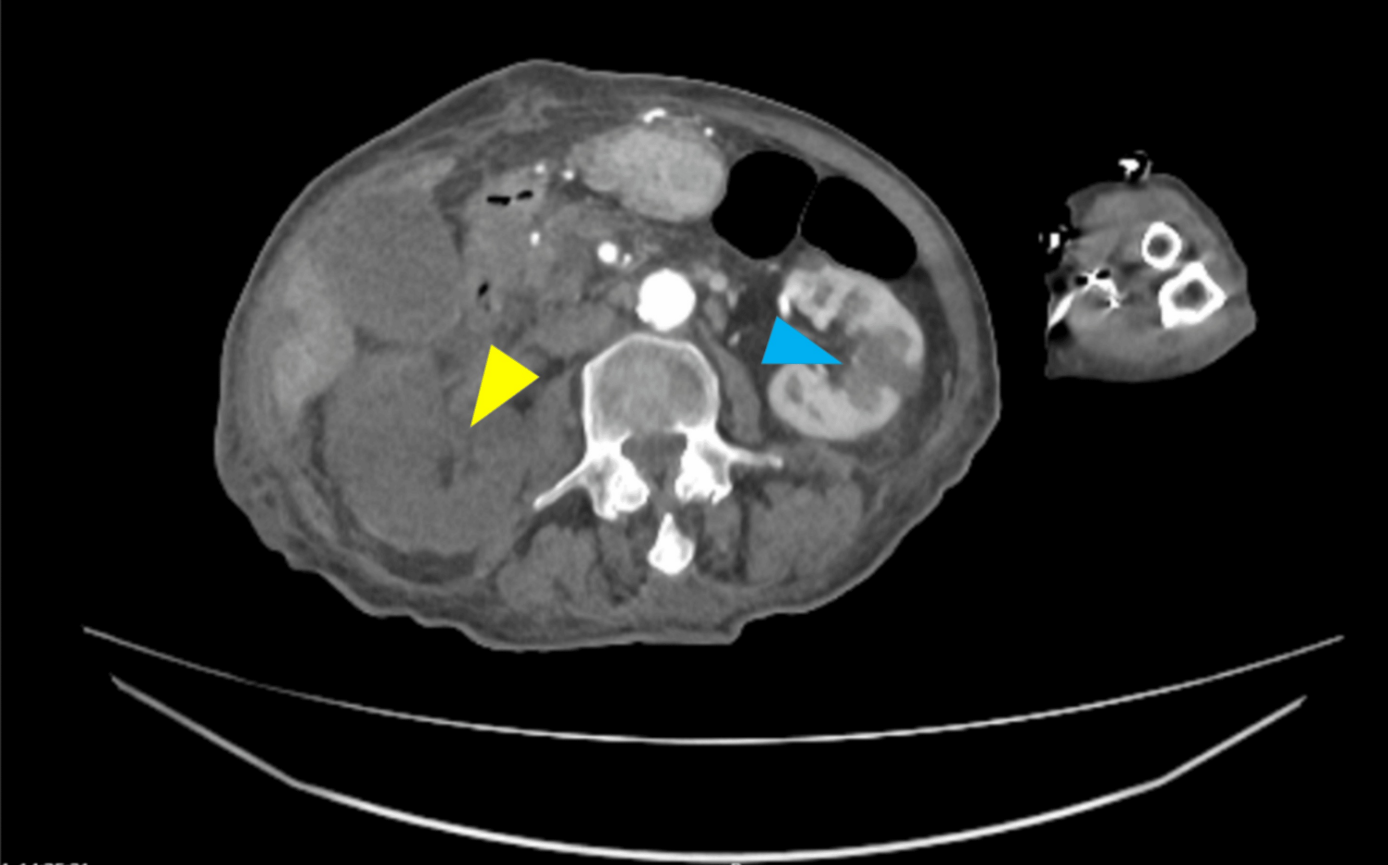
CT angiogram showing a complete lack of enhancement of the right kidney (yellow arrow) and segmental occlusion of the left kidney (blue arrow).

**Figure 3:**
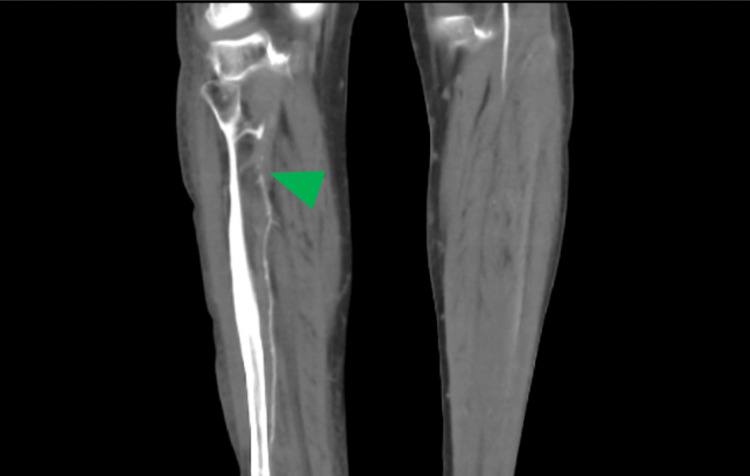
CT angiogram showing chronic long-segment stenosis of the anterior and posterior tibial arteries (green arrow), with acute embolic occlusion of the mid-to-distal right peroneal artery.

To investigate the suspected vascular compromise, a CT angiogram of the lower limbs was performed. The results were consistent with a central embolic shower. Key findings included the following: complete occlusion of the right proximal renal artery and multiple segmental infarcts within the left kidney and spleen (Figure 2); acute embolic occlusion of the mid-to-distal right peroneal artery, occurring alongside chronic long-segment stenosis of the anterior and posterior tibial arteries (Figure 3).

It was unknown if the patient was symptomatic of the chronic long-segment stenosis within the lower limbs.

Upon confirmation of multiorgan arterial infarction, systemic anticoagulation was prioritised to prevent further embolic events. Vascular surgeons were consulted for consideration of interventional or surgical management; however, given the poor prognosis and the patient's desire not to have further surgical intervention, the decision was made for conservative management. The patient was initially started on a weight-based unfractionated heparin (UFH) infusion following the CT angiogram, due to her renal impairment and in case of the need for surgical intervention. She was subsequently transitioned to therapeutic-dose LMWH after two days, with a plan for this to be continued for six weeks.

To definitively identify the embolic source, transthoracic echocardiography (TTE) was attempted. However, the examination could not be completed as the patient was unable to tolerate the positioning and physical discomfort required for the procedure. Transoesophageal echocardiography (TOE) was considered; however, the patient declined further invasive investigations.

The clinical findings, including the evidence of widespread arterial ischaemia and the advanced stage of the suspected malignancy, were discussed in a multidisciplinary setting, involving the patient and her family.

Recognising the poor prognosis associated with a central embolic shower and the frailty of her postoperative state, the patient expressed a clear preference for comfort-based management over further aggressive intervention. She declined additional diagnostic imaging, including TOE and biopsy.

Active treatment was ceased, and the patient was transitioned to a palliative care pathway, focused on symptom control. She was discharged to a local hospice for end-of-life care and died two days later. The rapid terminal decline in this case highlights the catastrophic nature of malignancy-induced arterial thromboembolism.

## Discussion

While malignancy-induced hypercoagulability typically manifests as venous thromboembolism (VTE), arterial events (though less frequent) carry a significantly higher morbidity and mortality. Pancreatic adenocarcinoma is associated with a high risk of VTE among solid organ tumours, especially within the first six months of diagnosis or during acute hospitalisation [3,5]. This case is uniquely characterised by a central embolic shower.

The primary suspected mechanism for this systemic distribution of arterial emboli is non-bacterial thrombotic endocarditis (NBTE). NBTE is a condition frequently associated with advanced mucin-producing carcinomas, such as those of the pancreas [6]. Given that approximately 80-90% of systemic emboli originate from a cardiac source [7], the presence of concurrent renal, splenic, and lower limb infarctions in this patient strongly suggests NBTE as the underlying driver.

The management of NBTE-related embolic showers is exceedingly difficult. While the standard of care remains systemic anticoagulation with heparin and treatment of the underlying malignancy, the prognosis for patients with multiorgan infarctions is often terminal. In this case, the patient's frailty and decision to decline further treatment prevented surgical or oncological intervention. Consequently, therapy was limited to conservative anticoagulation, which ultimately proved insufficient against the aggressive prothrombotic state.

Limitations

Diagnostic Challenges in Cardiac Imaging

A significant limitation of this report is the absence of definitive cardiac imaging. Although atrial myxoma is the most common benign cardiac tumour associated with systemic embolic showers [8,9], it was considered a less likely aetiology in this case, given the patient’s constitutional symptoms, weight loss, and the high clinical suspicion of underlying malignancy.

NBTE, often a manifestation of Trousseau’s syndrome in the setting of advanced adenocarcinoma, is a more probable cause for the multiorgan (bilateral renal and splenic) infarcts observed [10]. Unfortunately, the patient’s clinical instability and discomfort prevented the completion of a TTE. Furthermore, the patient declined a TOE, which remains the gold standard for identifying the small, friable vegetations typical of NBTE that are frequently missed by TTE. Consequently, while the clinical triad of malignancy, hypercoagulability, and systemic emboli is highly suggestive, a definitive diagnosis of NBTE or a primary cardiac tumour could not be established.

Absence of Histopathological Confirmation

An additional limitation is the lack of histological confirmation of the suspected pancreatic primary. While intraoperative visualisation of a pancreatic head mass and significantly elevated tumour markers (CA 19-9 levels >100,000 kU/L) provided a high degree of clinical certainty, the absence of a tissue biopsy prevents a definitive pathological grading or subtyping.

In clinical practice, the gold standard of tissue diagnosis must be weighed against the principle of non-maleficence. In the context of a rapidly deteriorating patient who had transitioned to a palliative pathway, the risk-to-benefit ratio of an invasive percutaneous or endoscopic ultrasound-guided biopsy was deemed unfavourable. The procedural risks precluded biopsy, as the results would not have altered the management plan focused on comfort and symptom control. This case highlights the occasional necessity of relying on clinical and radiological diagnosis in end-of-life care when diagnostic rigour conflicts with the patient’s goals of care.

## Conclusions

Malignancy-driven hypercoagulability presents a broad spectrum of thromboembolic challenges. While venous events are common, this case suggests that central embolic showers may occur in patients with advanced pancreatic malignancy, even in the absence of direct cardiac invasion. Given that pancreatic malignancy carries one of the highest risks of coagulopathy among solid organ tumours, clinicians should consider occult malignancy in the differential diagnosis of multiorgan infarcts, especially when cardiac sources are not immediately evident.

While this case has significant limitations, including the lack of histological diagnosis and definitive cardiac imaging, it provides pertinent learning points. The presence of simultaneous infarcts across multiple vascular beds should prompt urgent investigation for both central cardiac sources and occult underlying malignancy. Although systemic anticoagulation remains the cornerstone of management, the occurrence of central embolic showers often signifies advanced, progressive disease. Early recognition is therefore vital, not only for initiating therapy but for providing realistic prognostic guidance and facilitating timely goals-of-care discussions in these high-mortality clinical scenarios.
